# Supplementary material for: Hybrid cellular membrane nanovesicles amplify macrophage immune responses against cancer recurrence and metastasis
Source: Nat Commun. 2020 Sep 30;11:4909. doi: 10.1038/s41467-020-18626-y (PMC7527506; doi:10.1038/s41467-020-18626-y)
Supplement: Supplementary file 1 — Supplementary Information [file 41467_2020_18626_MOESM1_ESM.pdf]

Supplementary Information

Hybrid cellular membrane nanovesicles amplify macrophage immune responses against cancer recurrence and metastasis

Rao et al.

Contents

Supplementary Figures S2

Supplementary Tables S24

Supplementary Methods S25

Supplementary References S30

Supplementary Figures

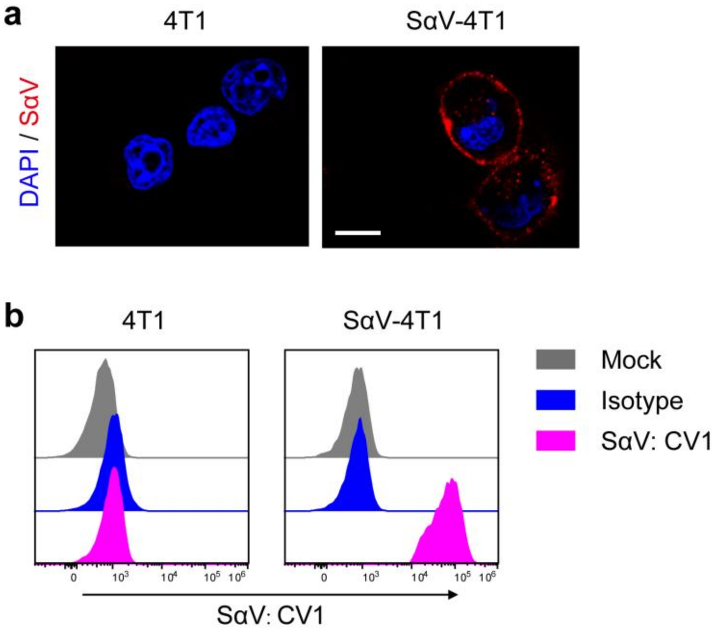

Supplementary Figure 1. SIRPα variant expression verification. **a,b,** Immunofluorescence imaging (**a**) and flow cytometry analysis (**b**) of SIRPα variant (SαV; CV1) expression on original and engineered 4T1 cells. Scale bar, 10 µm.

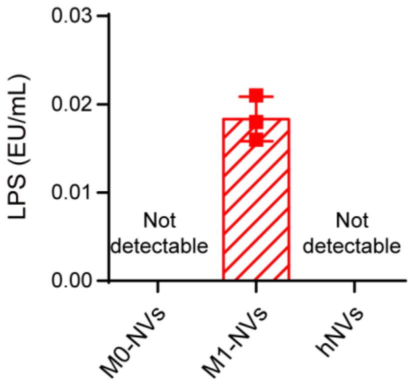

Supplementary Figure 2. Detection of LPS contents in M0-NVs, M1-NVs and hNVs. All data are presented as mean ± S.D. (n = 3).

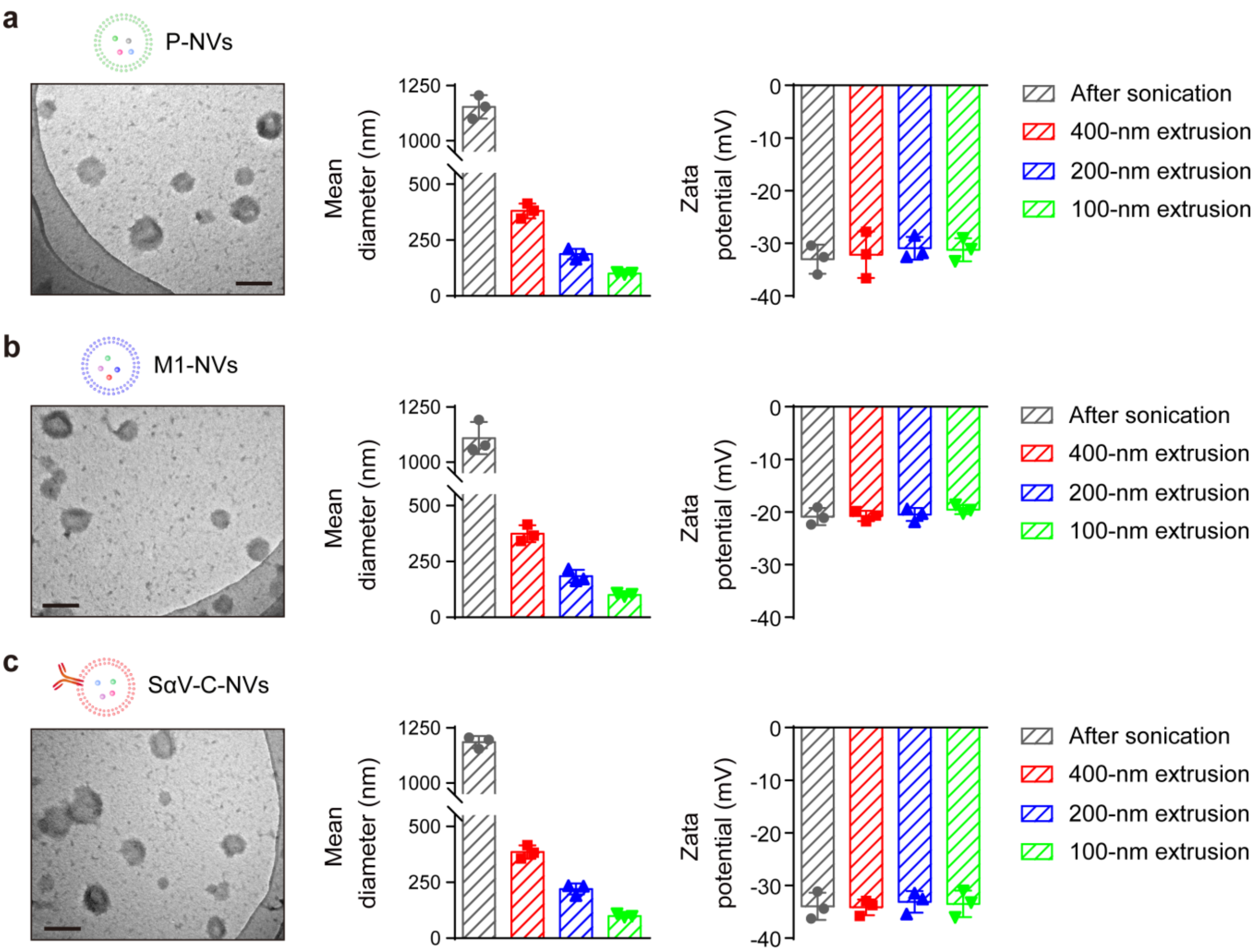

Supplementary Figure 3. Preparation and characterization of various NVs. TEM images, mean diameter and zeta potential of P-NVs (**a**), M1-NVs (**b**) and SαV-C-NVs (**c**) following sonication and extrusion through membranes. Scale bars, 100 nm. The samples were negatively stained with uranyl acetate. All data are presented as mean ± S.D. (n = 3).

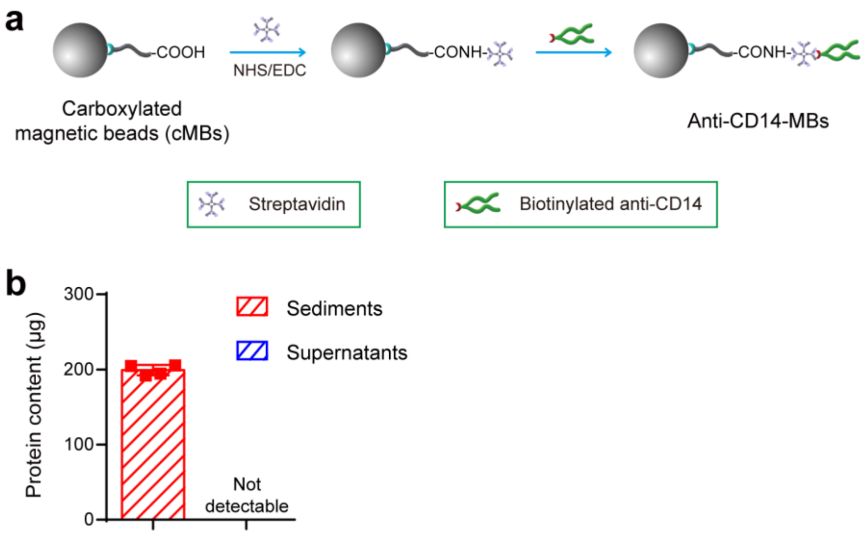

Supplementary Figure 4. Purification of the hNVs by pull down assay with the anti-CD14 antibody-modified magnetic beads. **a**, The design of the immune magnetic beads for the pull down assay. **b,** Measurement of the protein content in the sediments and supermatants of hNVs after the pull down assay. All data are presented as mean ± S.D. (*n* = 4).

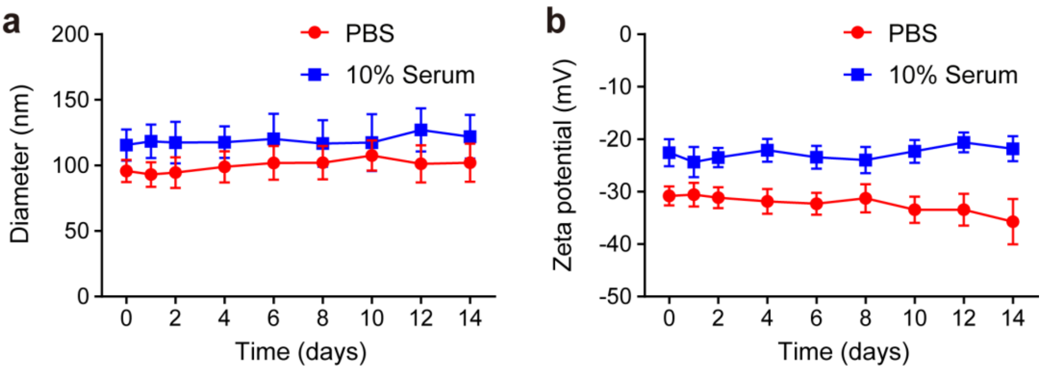

Supplementary Figure 5. Stability of hNVs. Mean diameter (**a**) and zeta potential (**b**) of hNVs in PBS and 10% serum over 2 weeks. All data are presented as mean ± S.D. (n = 3).

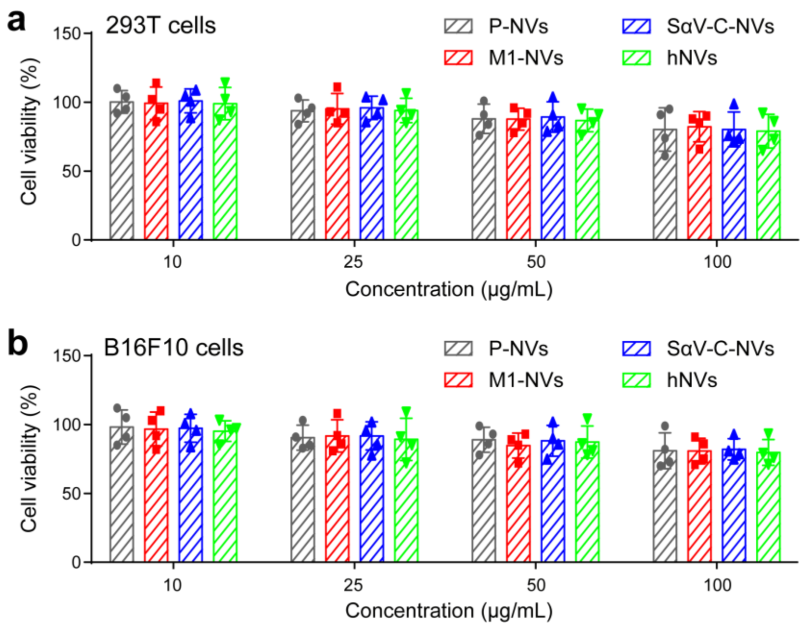

Supplementary Figure 6. *In vitro* biocompatibility of various NVs. Human embryonic kidney 293T cell (**a**) and murine melanoma B16F10 cell (**b**) viability after incubation with P-NVs, M1-NVs, SαV-C-NVs and hNVs for 48 h. All data are presented as mean ± S.D. (n = 4).

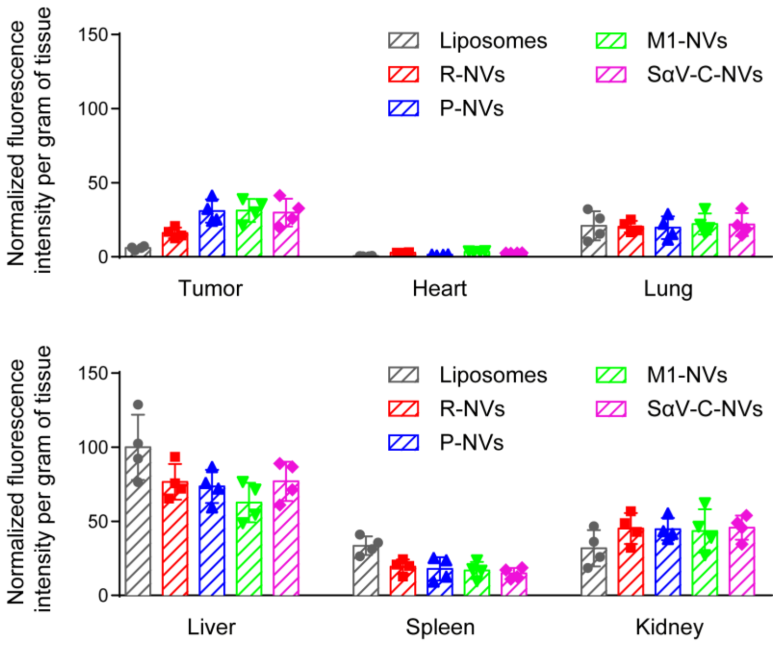

Supplementary Figure 7. *In vivo* distribution of liposomes and various NVs in tumor tissues and major organs including heart, lung, liver, spleen and kidney. All data are presented as mean ± S.D. (n = 4).

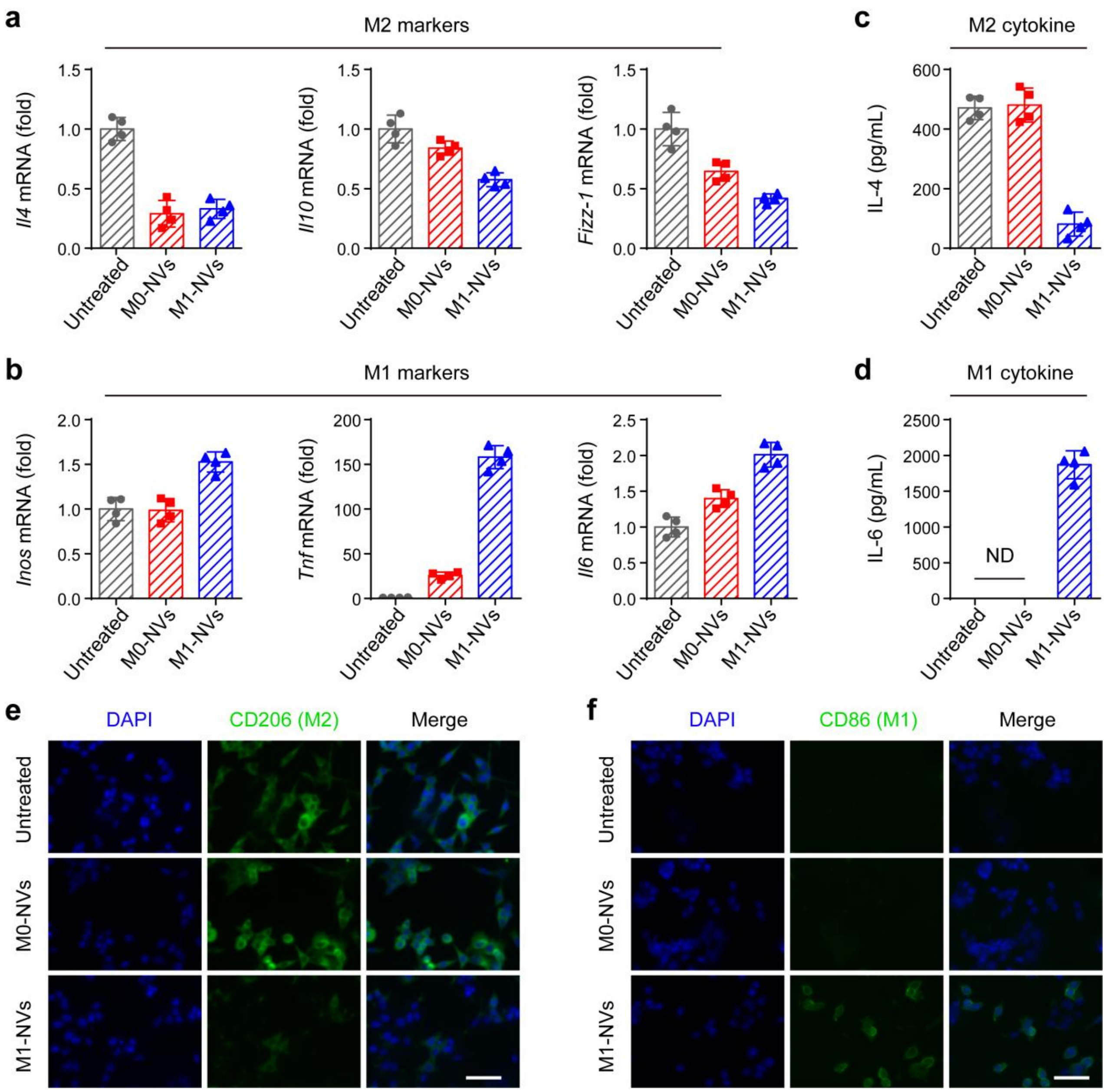

Supplementary Figure 8. *In vitro* repolarization of M2 macrophages to M1 ones by M1-NVs. **a**,**b**, Relative mRNA expressions of *Il4*, *Il10*, Fizz-1 (M2 marker; **a**) and *Inos, Tnf*, *and Il6* (M1 marker; **b**) in M2 macrophages treated with either M0-NVs or M1-NVs for 24 h. **c**,**d**, ELISA measurement of the secretion of anti-inflammatory cytokine IL-4 (M2 marker; **c**) and pro-inflammatory cytokine IL-6 (M1 marker; **d**) from M2 macrophages treated with either M0-NVs or M1-NVs for 24 h. ND denotes no detection. **e**,**f**, Immunofluorescence staining for CD206 (green, M2 marker; **e**) and CD86 (green, M1 marker; **f**) of M2 macrophages treated with either M0-NVs or M1-NVs for 24 h. Scale bars, 50 μm. The cell nuclei were stained with DAPI (blue). All data are presented as mean ± S.D. (*n* = 4).

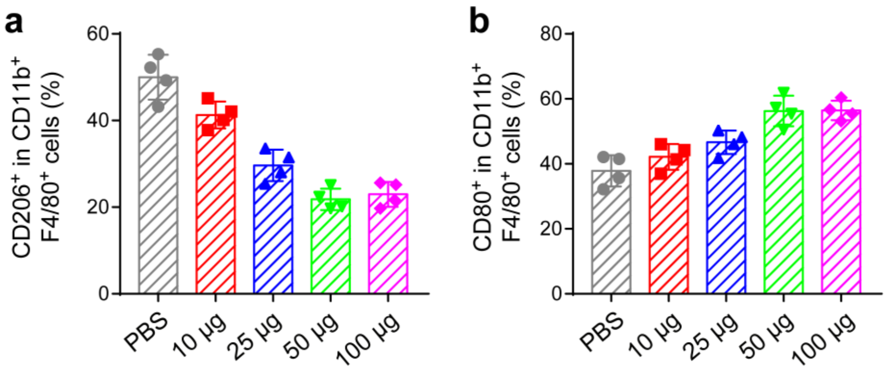

Supplementary Figure 9. *In vivo* dose effects of M1-NVs on macrophage polarization. **a**,**b**, Flow cytometry analysis of M2-type macrophages ($CD206^+$; **a**) and M1 ones ($CD80^+$; **b**) after treatment with different amounts of M1-NVs. Data are presented as mean ± S.D. (n = 4).

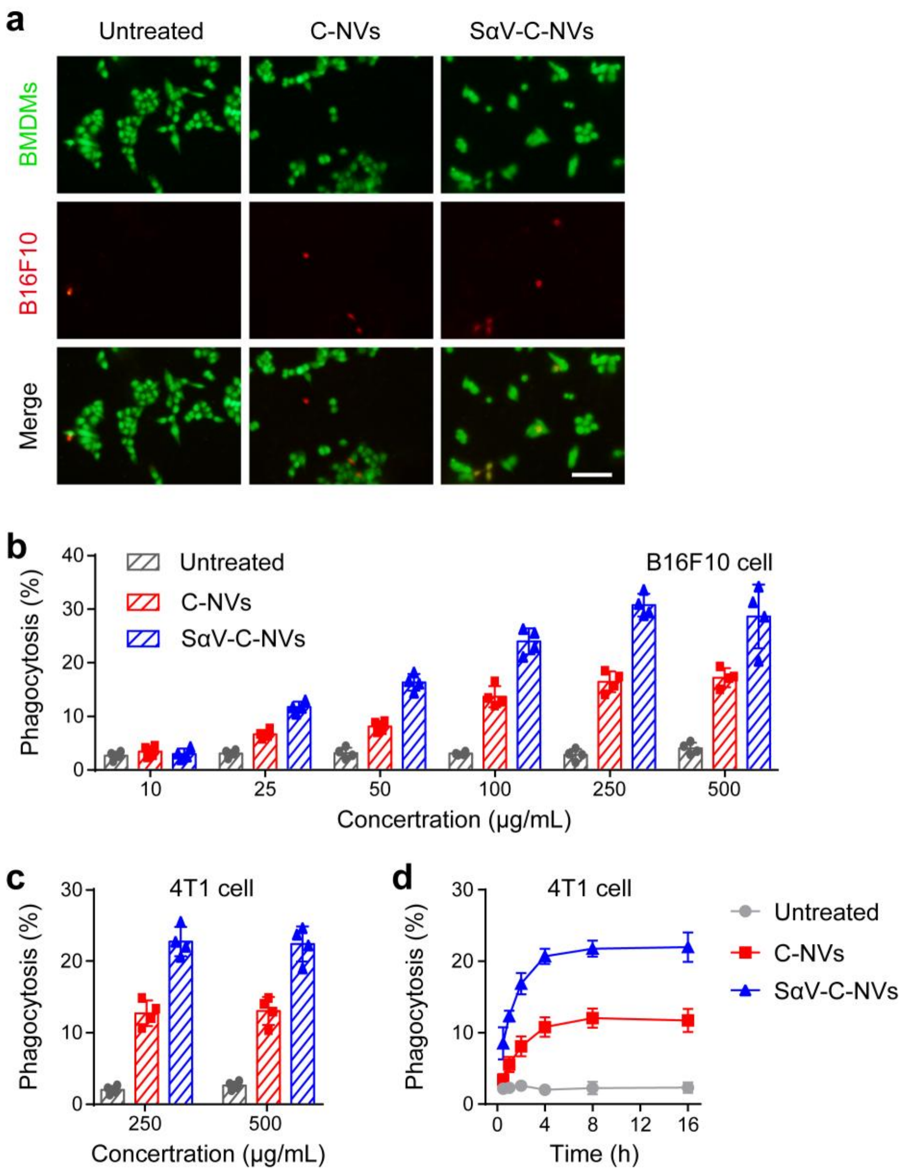

Supplementary Figure 10. *In vitro* CD47 blockade by SαV-C-NVs increases the phagocytosis of cancer cells by macrophages. **a**, Representative confocal images of phagocytosis assays, in which B16F10 cancer cells were labelled with CellTracker DeepRed (red) and bone-marrow-derived macrophage cells (BMDMs) were labelled with CellTracker Green (green). Scale bar, 150 μm. **b**, Quantification analysis of the phagocytosis of B16F10 cancer cells by BMDMs. **c**,**d**, Quantification analysis of the phagocytosis of 4T1 cancer cells by BMDMs with different concentrations (**c**) or different incubation time periods (**d**). All data are presented as mean ± S.D. (n = 4).

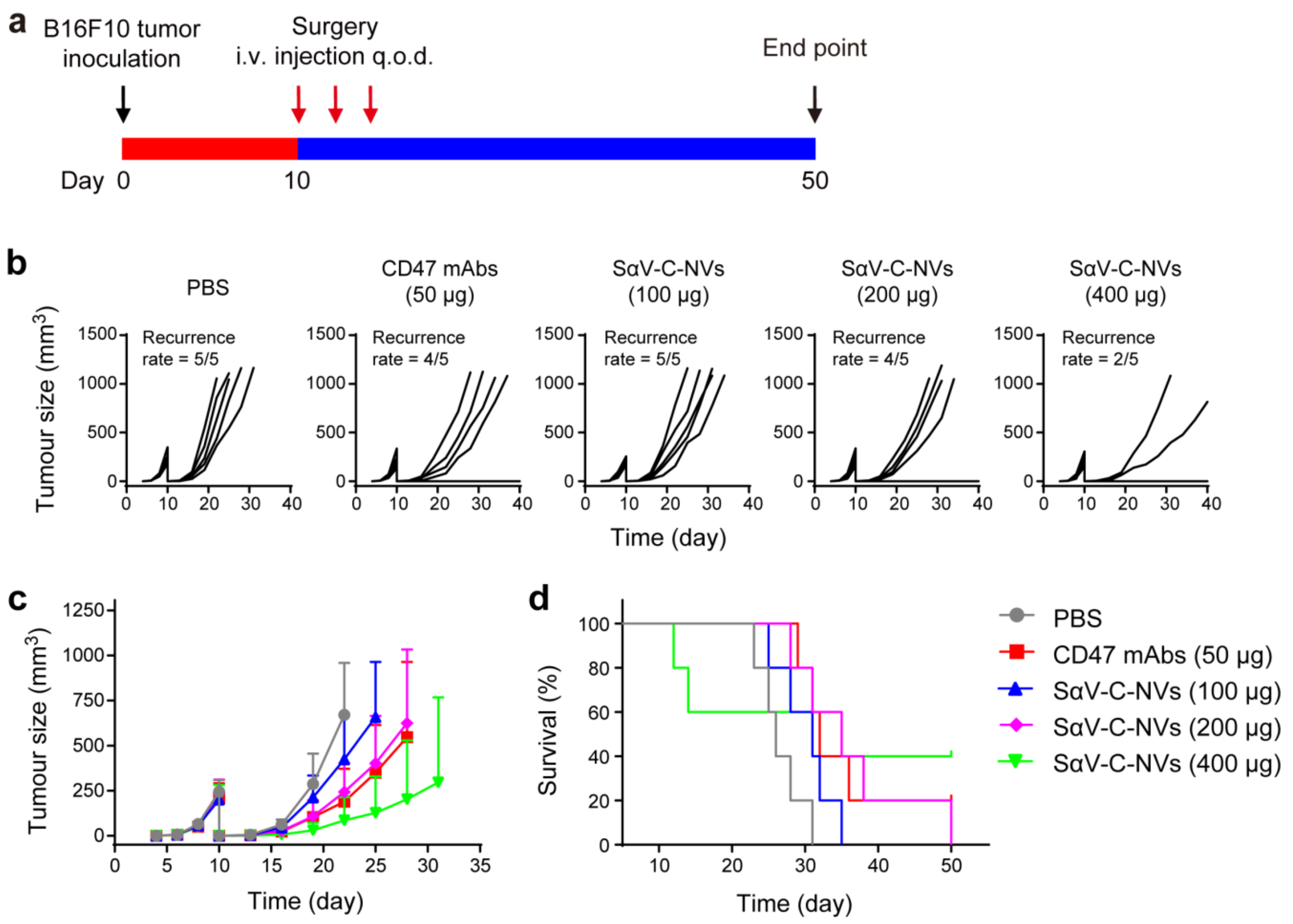

Supplementary Figure 11. *In vivo* dose effects of hNVs on inhibiting post-surgery recurrence of B16F10 tumors. **a**, Schematic showing the treatment schedule in a recurrence mouse model after incomplete surgery. **b**,**c**, Individual (**b**) and average (**c**) tumor growth kinetics in different groups. Growth curves were stopped when the first mouse of the corresponding group died. **d**, Survival corresponding to the tumor size of mice after different treatments as indicated. All data are presented as mean ± S.D. (*n* = 5).

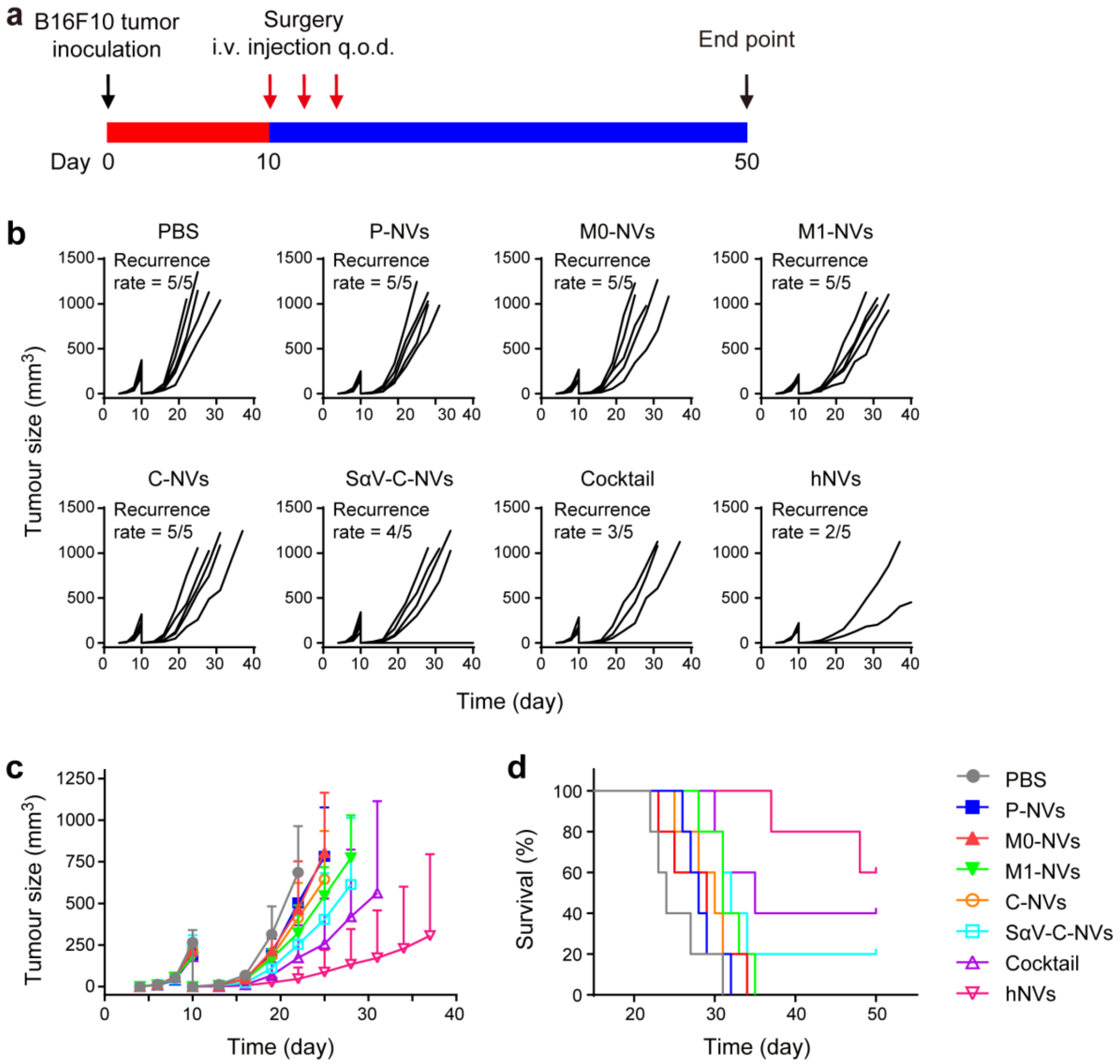

Supplementary Figure 12. *In vivo* effects of normalized dose NVs on inhibiting post-surgery recurrence of B16F10 tumors. **a**, Schematic showing the treatment schedule in a recurrence mouse model after incomplete surgery. **b**,**c**, Individual (**b**) and average (**c**) tumor growth kinetics in different groups. Growth curves were stopped when the first mouse of the corresponding group died. **d**, Survival corresponding to the tumor size of mice after different treatments as indicated. All data are presented as mean ± S.D. (*n* = 5).

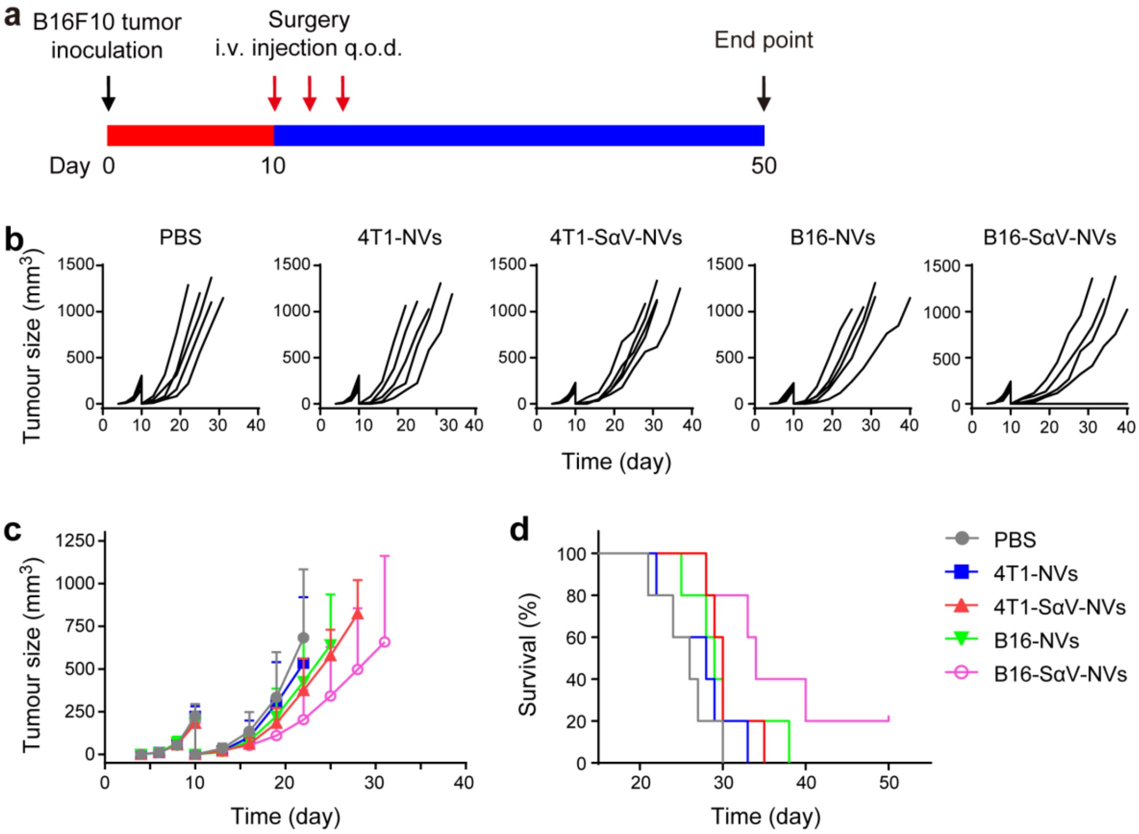

Supplementary Figure 13. *In vivo* effects of B16F10- or 4T1-derived NVs on inhibiting post-surgery recurrence of B16F10 tumors. **a**, Schematic showing the treatment schedule in a recurrence mouse model after incomplete surgery. **b**,**c**, Individual (**b**) and average (**c**) tumor growth kinetics in different groups. Growth curves were stopped when the first mouse of the corresponding group died. **d**, Survival corresponding to the tumor size of mice after different treatments as indicated. All data are presented as mean ± S.D. (*n* = 5).

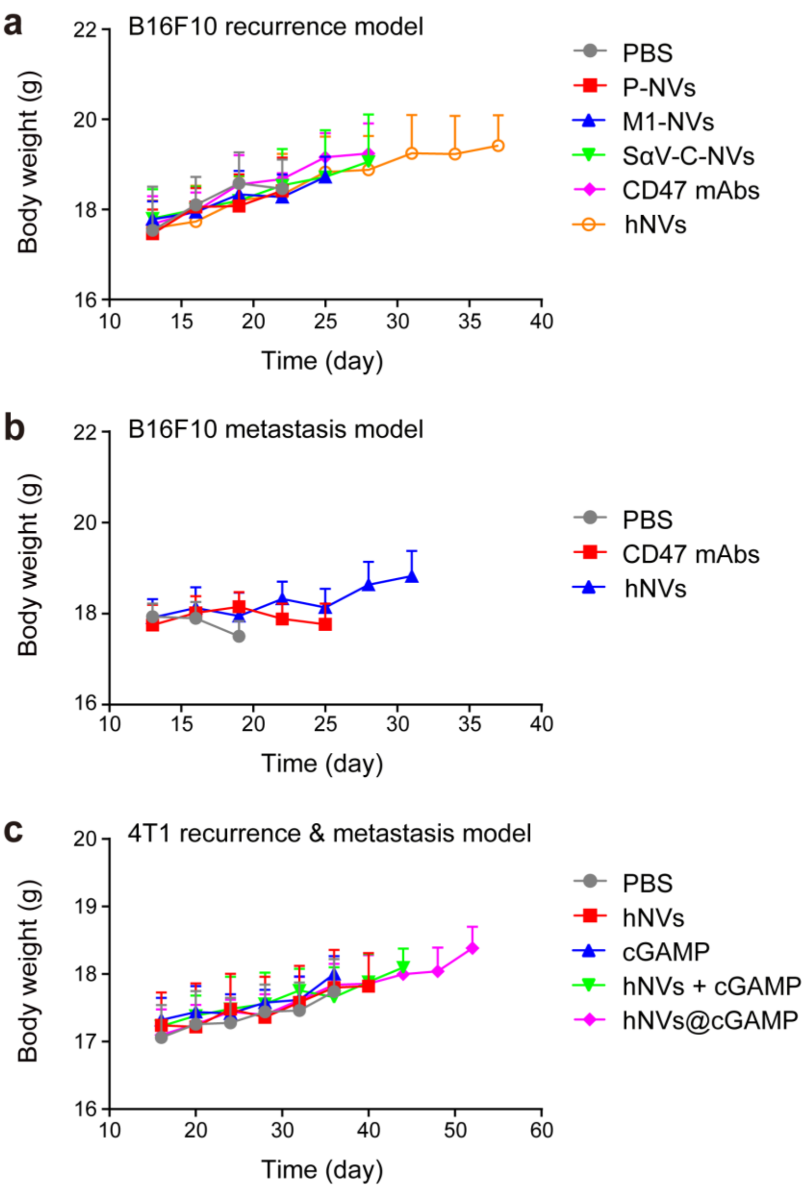

Supplementary Figure 14. Mice body weight curves in the B16F10 malignant melanin recurrence model (Figure 3; **a**), B16F10 metastasis model (Figure 5; **b**), and 4T1 triple negative breast carcinoma model (Figure 6; **c**), respectively. All data are presented as mean ± S.D. (**a**, *n* = 6 for the hNVs-treated group, *n* = 5 for the other groups; **b**, *n* = 8 for the hNVs-treated group, *n* = 6 for the other groups; **c**, *n* = 7 for the groups treated with hNVs + cGAMP and hNVs@cGAMP, *n* = 5 for the other groups).

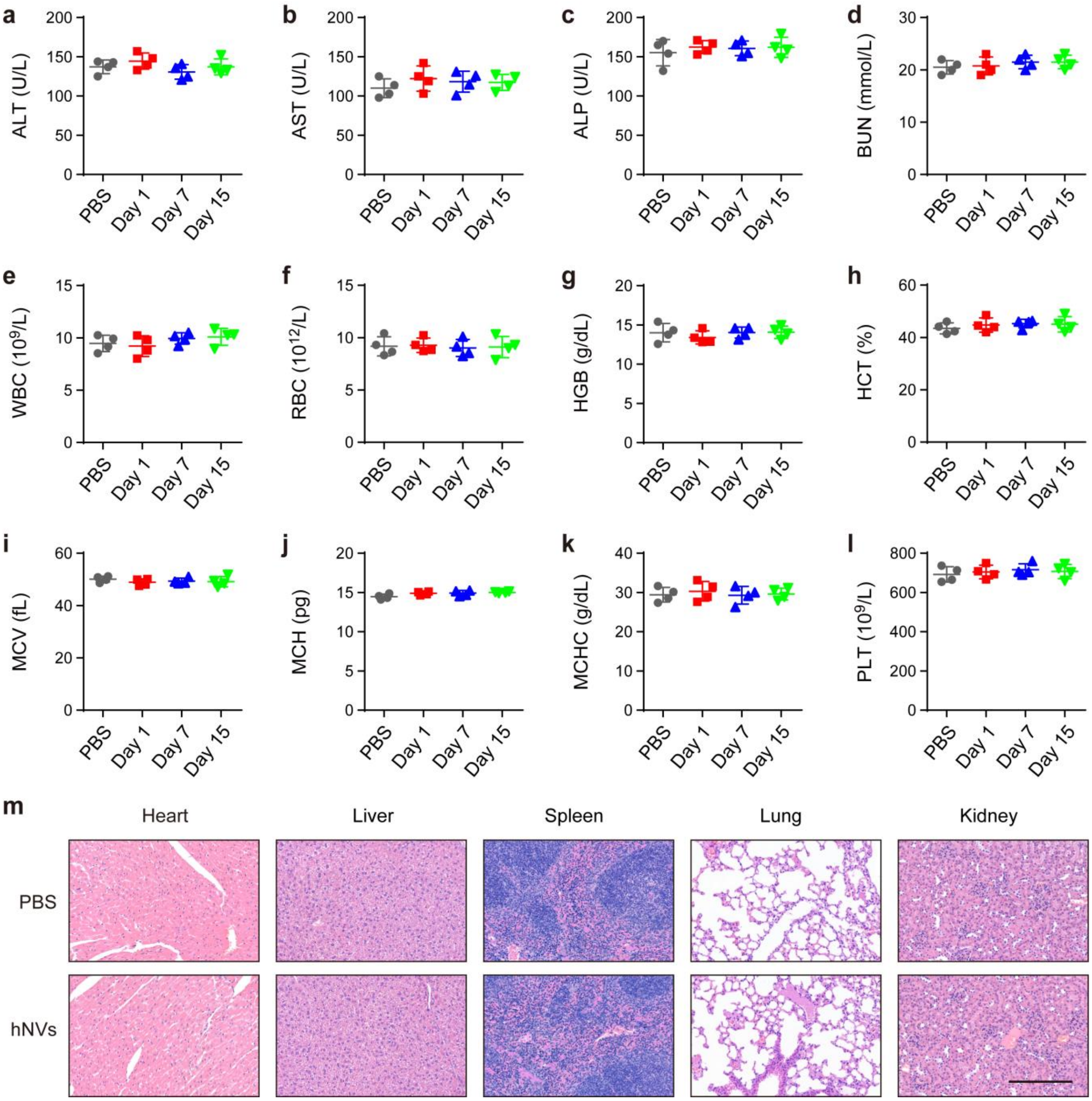

Supplementary Figure 15. *In vivo* toxicity evaluation. **a**, ALT: alanine transaminase. **b**, AST: aspartate aminotransferase. **c**, ALP: alkaline phosphatase. **d**, BUN: blood urea nitrogen. **e**, WBC: white blood cell. **f**, RBC: red blood cell. **g**, HGB: hemoglobin. **h**, HCT: hematocrit. **i**, MCV: mean corpuscular volume. **j**, MCH: mean corpuscular hemoglobin. **k**, MCHC: mean corpuscular hemoglobin concentration. **l**, PLT: platelets. **m**, H&E-stained slice images of major organs. Scale bar, 200 μm. The mice received repeated i.v. injections with PBS or hNVs every other day and the blood collected from mice at day 1, 7, and 15 post the injection were used for blood biochemistry and complete blood panel analysis. At day 30 post the injection, major organs were collected from mice for histology analysis. All data are presented as mean ± S.D. (n = 4).

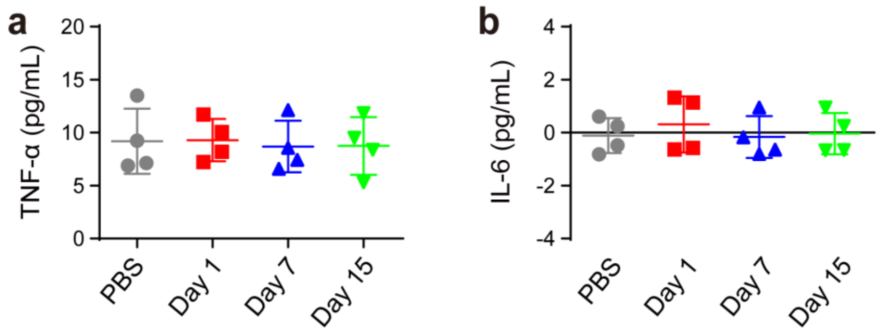

Supplementary Figure 16. Serum cytokine measurement. **a**, TNF-α: tumor necrosis factor-α. **b**, IL-6: interleukin-6. The mice received repeated i.v. injections with PBS or hNVs every other day and the blood collected from mice at day 1, 7, and 15 post the injection were used for ELISA measurement of cytokines. All data are presented as mean ± S.D. (n = 4).

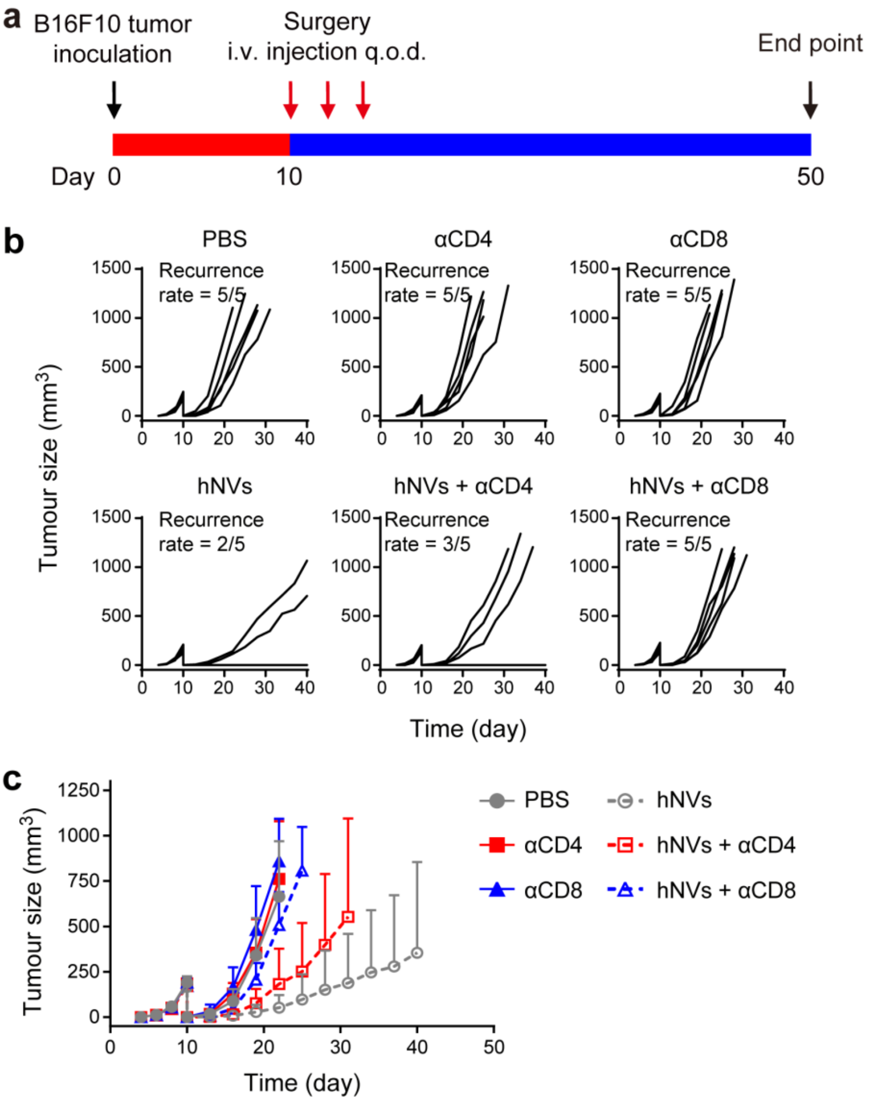

Supplementary Figure 17. *In vivo* effects of $CD4^+$ and $CD8^+$ T cells on inhibiting post-surgery recurrence of B16F10 tumors. **a**, Schematic showing the treatment schedule in a recurrence mouse model after incomplete surgery. **b**,**c**, Individual (**b**) and average (**c**) tumor growth kinetics in different groups. Growth curves were stopped when the first mouse of the corresponding group died. All data are presented as mean ± S.D. (n = 5).

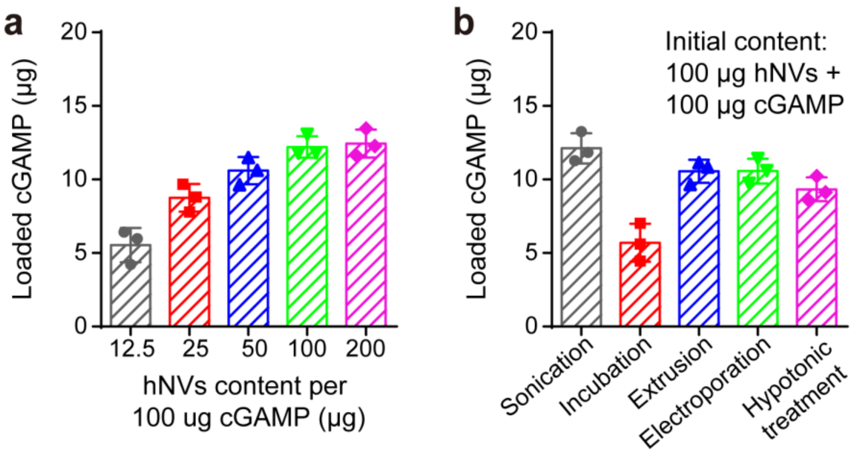

Supplementary Figure 18. Drug loading efficiency in hNVs. **a**, Drug loading content of hNVs with different ratios of cGAMP to hNVs by sonication. **b,** Drug loading content of hNVs by different methods. All data are presented as mean ± S.D. (*n* = 3).

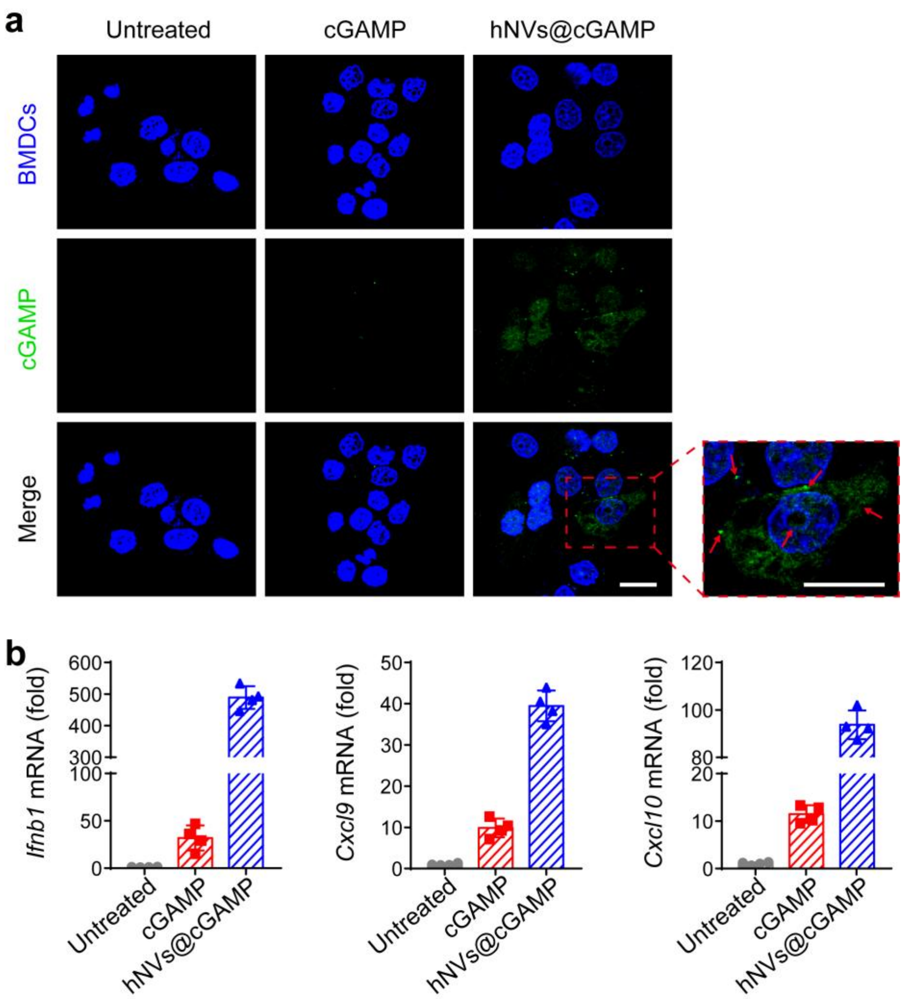

Supplementary Figure 19. hNVs for enhanced cytosolic delivery of cGAMP. **a**, Representative confocal images of bone marrow-derived dendritic cells (BMDCs) after incubation with fluorescein-conjugated cGAMP in free or hNVs form. Scale bars, 25 µm. **b**, Relative mRNA expressions of *Ifnb1*, *Cxcl9* and *Cxcl10* in BMDCs after indicated treatments. All data are presented as mean ± S.D. (n = 4).

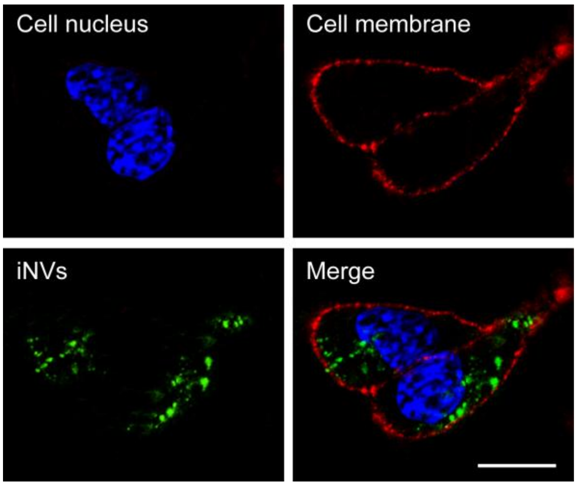

Supplementary Figure 20. Immunofluorescence imaging of the entry of hNVs into B16F10 cells. Scale bar, 10 µm. Before the confocal imaging, the cell nucleus, cell membrane and hNVs were labeled with DAPI, DiD and DiO, respectively.

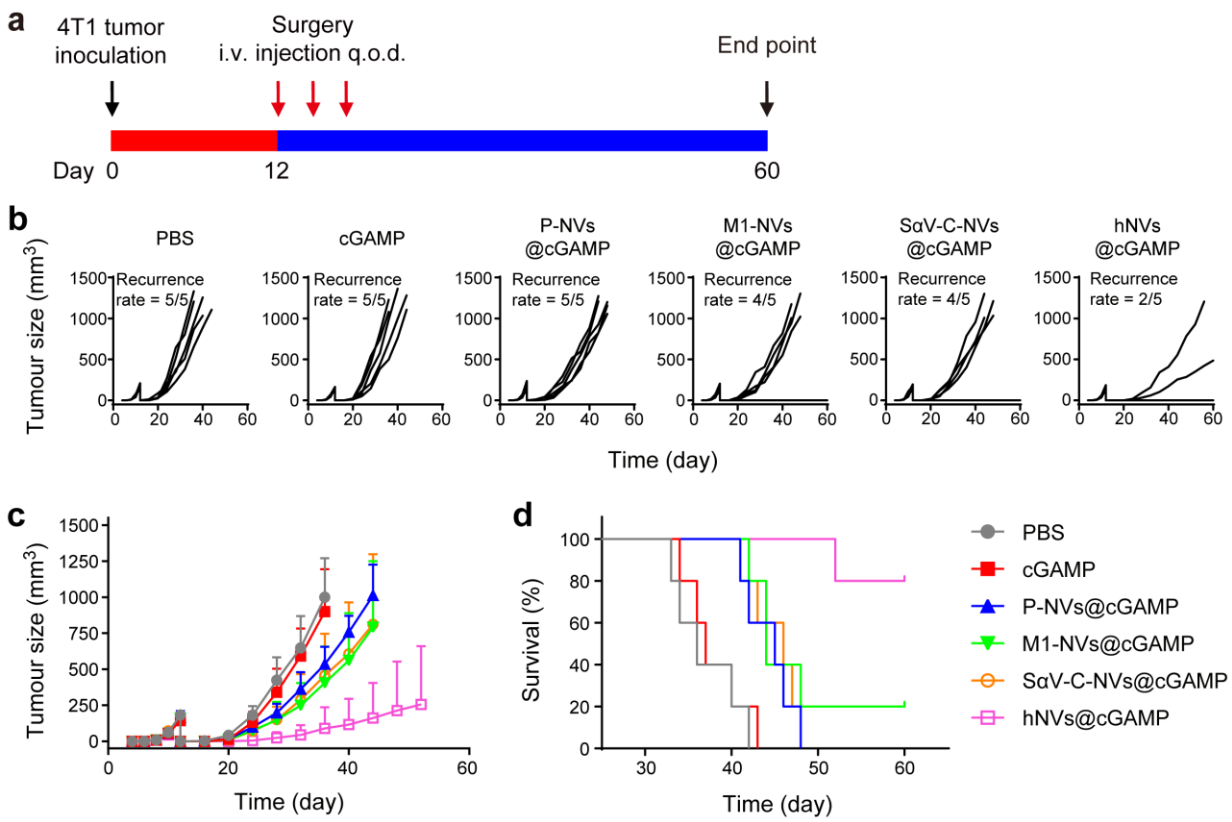

Supplementary Figure 21. *In vivo* effects of various NVs loaded with cGAMP on inhibiting 4T1 tumors. **a**, Schematic showing the treatment schedule in a recurrence mouse model after incomplete surgery. **b**,**c**, Individual (**b**) and average (**c**) tumor growth kinetics in different groups. Growth curves were stopped when the first mouse of the corresponding group died. **d**, Survival corresponding to the tumor size of mice after different treatments as indicated. All data are presented as mean ± S.D. (n = 5).

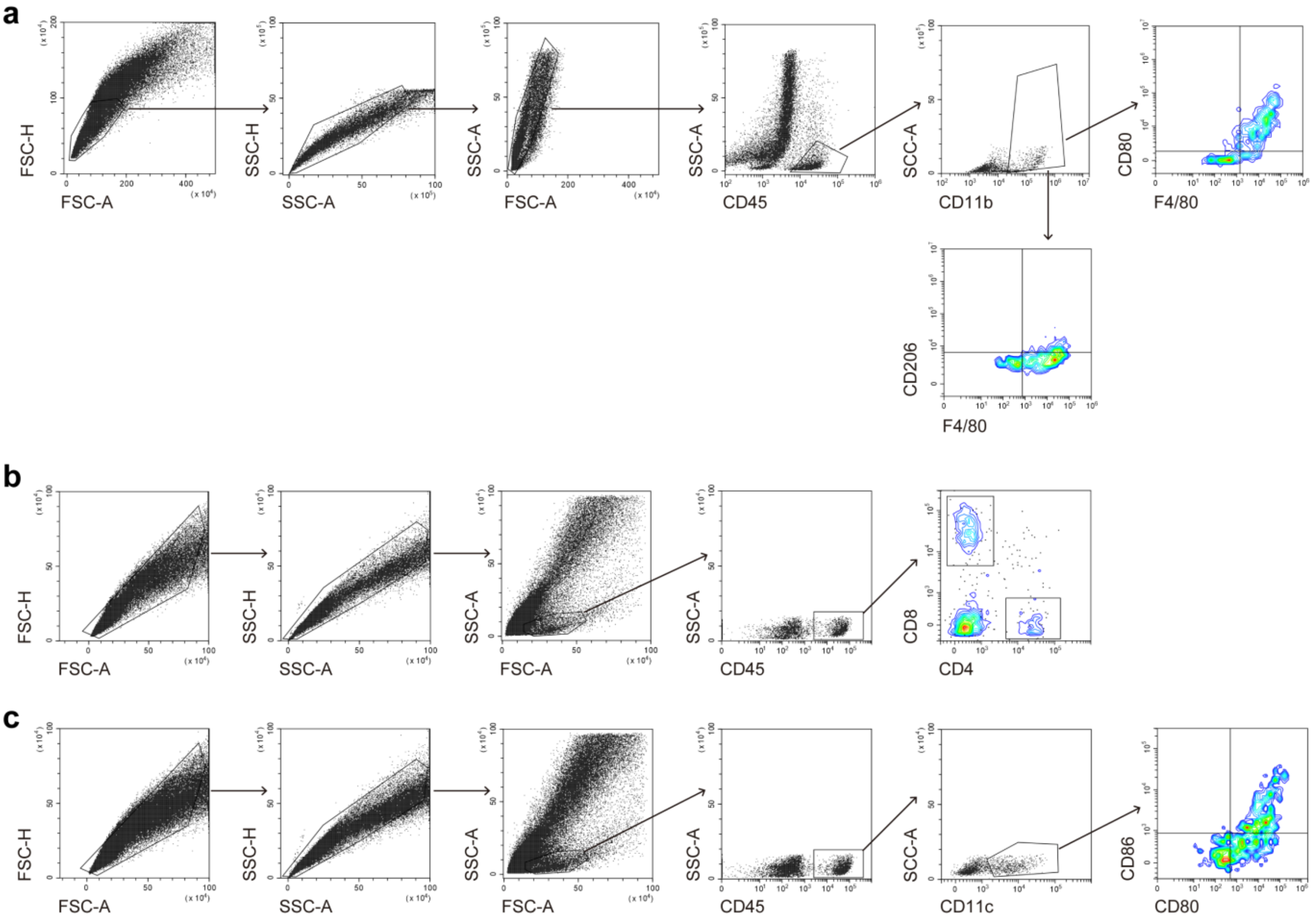

Supplementary Figure 22. Graphically account for flow cytometry gating strategies. **a**, Gating strategy to sort CD206$^+$ M2-like and CD80$^+$ M1-like macrophages in tumor tissues gating on F4/80$^+$CD11b$^+$CD45$^+$ cells presented on Figs. 2h and 4a. **b**, Gating strategy to sort CD8$^+$ and CD4$^+$ T cells in tumor tissues gating on CD45$^+$ cells presented on Figs. 2k and 4b. **c**, Gating strategy to sort CD80$^+$CD86$^+$ dendritic cells in tumor tissues gating on CD45$^+$CD11c$^+$ cells presented on Fig. 2i.

Supplementary Tables

Supplementary Table 1. All primer sequences used in this work.

Cd86 Forward	5'-GATTATCGGAGCGCCTTTCT-3'
Cd86 Reverse	5'-CCACACTGACTCTTCCATTCTT-3'
Tnf Forward	5'-CCTGTAGCCCACGTCGTAGC-3'
Tnf Reverse	5'-AGCAATGACTCCAAAGTAGACC-3'
Il4 Forward	5'-ACTTGAGAGAGATCATCGGCATTT-3'
Il4 Reverse	5'-AGCACCTTGGAAGCCCTACAG-3'
Il6 Forward	5'-ACAAAGCCAGAGTCCTTCAGAGA-3'
Il6 Reverse	5'-CTGTTAGGAGAGCATTGGAAATTG-3'
Il10 Forward	5'-ACTGGCATGAGGATCAGCAG-3'
Il10 Reverse	5'-CTCCTTGATTTCTGGGCCAT-3'
Fizz-1 Forward	5'-AGGATGCCAACTTTGAATAGGA-3'
Fizz-1 Reverse	5'-CGAGTAAGCACAGGCAGTT-3'
Inos Forward	5'-TCACCTTCGAGGGCAGCCGA-3'
Inos Reverse	5'-TCCGTGGCAAAGCGAGCCAG-3'
Ifnb1 Forward	5'-CTGGCTTCCATCATGAACAA-3'
Ifnb1 Reverse	5'-AGAGGGCTGTGGTGGAGAA-3'
Cxcl9 Forward	5'-CAGCTCTGCCATGAAGTCCG-3'
Cxcl9 Reverse	5'-AGGGTTCCTCGAACTCCACAC-3'
Cxcl10 Forward	5'-TCATCCTGCTGGGTCTGAGT-3'
Cxcl10 Reverse	5'-CATCGTGGCAATGATCTCAACA-3'

Supplementary Methods

In vitro biocompatibility assay

CCK-8 assay was used to evaluate the biocompatibility of NVs *in vitro*[1]. Briefly, 293T and B16F10 cells were seeded in 96-well plates at a density of 1×10^4 cells per well and cultured for 12 h. Then different concentrations of P-NVs, M1-NVs, SαV-C-NVs, or hNVs were added to the medium, and cells were incubated for 48 h. Cells grown without any particles were used as a control. At the end of the incubation, CCK-8 was added to test the cell viability.

In vitro analysis of macrophage polarization by M1-NVs

M2 macrophages were induced by treating bone marrow-derived macrophages (BMDMs) with 20 ng/mL IL-4 (Invitrogen). Concentrations of 50 ng/mL of either M0-NVs or M1-NVs were used to treat M2 macrophages for 24 h for repolarization. Moreover, qPCR was performed to determine the expressions of M2- (IL-4, IL-10, and Fizz-1) and M1-associated genes (IL-6, iNOS, and TNF-α), as mentioned earlier. To confirm the production of the M1 marker IL-6 and suppression of the M2 marker IL-4 by M1-NVs. M2 macrophages were treated with 50 μg/mL M1-NVs for 24 h and the cytokine secretion was analyzed using mouse IL-6 and IL-4 ELISA kits (R&D Systems) following the manufacturer’s instructions. The protein expressions of the M1 macrophage marker (CD86) and M2 macrophage marker (CD206) were evaluated by an immunocytochemistry assay. Cells were fixed with 4% paraformaldehyde for 10 min at room temperature and washed in PBS. Primary antibodies against CD86 (Santa Cruz Biotechnology) and CD206 (Abcam) were used for staining. The samples were then incubated in PBS containing rhodamineconjugated secondary antibodies (Jackson-Immunoresearch) for 1 h at room temperature. All samples were mounted with mounting solution containing DAPI (Vector Laboratories) to stain the nuclei and were imaged using a fluorescent microscope.

***In vivo* dose-dependent evaluation of M1-NVs**

To compare the *in vivo* effects of different concentrations of M1-NVs on the repolariztion of TAM, B16F10 tumor recurrence model was used. After surgery, the mice received three doses of i.v. injection of PBS, or different concentrations M1-NVs (*i.e.*, 10, 25, 50 or 100 µg per mouse) every other day. On 5th day after the last injection, tumor tissues were collected for flow cytometric analysis of M2-like TAMs (CD206^{+}CD11b^{+}F4/80^{+}) and M1-like TAMs (CD80^{+}CD11b^{+}F4/80^{+}) as described previously.

***In vitro* analysis of macrophage phagocytosis by SαV-C-NVs**

For fluorescence microscopic analysis, B16F10 or 4T1 cells were first treated with different concentrations of SαV-C-NVs. And then, BMDMs stained with CellTracker™ Green (Thermo Fisher Scientific) were seeded to 35 mm glass bottom dish at a density of 5 × 10^{5}, and co-cultured with 1 × 10^{6} of treated B16F10 or 4T1 cells stained with CellTracker™ Red (Thermo Fisher Scientific). After different time periods of co-incubation, phagocytosis of cancer cells by macrophages was analyzed as red positive signal related with phagosome formation in BMDMs.

***In vivo* dose-dependent evaluation of SαV-C-NVs**

To compare the *in vivo* effects of different concentrations of SαV-C-NVs and CD47 mAbs on the inhibition of tumor growth, B16F10 tumor recurrence model was used. 1 × 10^{6} luciferase-tagged B16F10 cancer cells were first transplanted into the right flank of C57BL/6. On 10th day after the inoculation, the tumors were resected, leaving about 1% residual tumor tissue behind to mimic the presence of residual microtumors in the surgical bed. The wound was closed using an Autoclip wound closing system. After surgery, the mice received three doses of i.v. injection of PBS, CD47 mAbs (50 µg per mouse) or different concentrations SαV-C-NVs (*i.e.*, 50, 100, 200 or 400 µg per mouse) every other day. Tumor volume was measured with a digital calliper. Animals were

euthanized when exhibiting signs of impaired health or when the volume of the tumor exceeded 1.5 cm^3.

***In vivo* evaluation of normalized dose NVs**

B16F10 tumor recurrence model was developed as described above. After surgery, the mice received three doses of i.v. injection of PBS, P-NVs, M0-NVs, M1-NVs, C-NVs, SαV-C-NVs, cocktail (simple mixture of P-NVs, M1-NVs, and SαV-C-NVs) and hNVs (200 μg per mouse for all NVs) every other day. Tumor volume was measured and animals were treated as described above.

***In vivo* evaluation of B16F10- or 4T1-derived NVs**

B16F10 tumor recurrence model was developed as described above. After surgery, the mice received three doses of i.v. injection of PBS, 4T1-NVs, 4T1-SαV-NVs, B16-NVs, and B16-SαV-NVs (200 μg per mouse) every other day. Tumor volume was measured and animals were treated as described above.

***In vivo* evaluation of $CD4^+$ and $CD8^+$ T cells on antitumor effects**

B16F10 tumor recurrence model was developed as described above. After surgery, the mice received three doses of i.v. injection of PBS and hNVs (200 μg per mouse) every other day. For $CD4^+$ and $CD8^+$ T cell depletion experiments, 300 mg anti-CD4 or anti-CD8 mAb was delivered 5 times by i.p. injection every 3 days.[2] Tumor volume was measured and animals were treated as described above.

***In vivo* toxicity evaluation**

In order to test the potential *in vivo* toxicity of hNVs, C57BL/6 mice received three doses of i.v. injection of PBS or hNVs (300 μg per mouse) every other day. General status of the mice was evaluated every day by veterinarian. Complete blood panel test and serum biochemistry assay

conducted at 1, 7 and 15 days after the treatment. Also, TNF-α and IL-6 levels in the serum was measured by mouse TNF-α and IL-6 ELISA Quantitation Kit (eBioscience). All mice were euthanized on the 30th day after the injection and their major organs (*i.e.*, hearts, livers, spleens, lungs and kidneys) were collected. Part of organs were fixed in 4% neutral buffered formalin, processed routinely into paraffin and sectioned at 4 μm. Then the sections were stained with H&E, and examined using an optical microscope.

cGAMP loading

The loading of cGAMP into the hNVs was achieved as follows: 100 μg cGAMP was mixed with different amounts of hNVs (*i.e.*, 12.5, 25, 50, 100 and 200 μg) and sonicated for 20 min at room temperature.. The hNVs@cGAMP were centrifuged and repeated washing with PBS to remove free cGAMP. All the washing solutions were collected, and the concentration of cGAMP was measured by UV-Vis spectrometer. The drug loading efficiency (DLE) was calculated from the difference in the concentration of the initial and left cGAMP in the supernatant. Side by side comparison of sonication, incubation, extrusion, electroporation and hypotonic treatment on the cGAMP loading was further carried out.

***In vitro* evaluation of cytosolic delivery of cGAMP by hNVs**

Bone marrow-derived dentritic cells (BMDCs) are generated according to the protocol we recently reported[3]. Briefly, bone marrow cells were collected from leg bones of mice. The cells were placed and cultured in a 24-well plate with complete RPMI 1640 medium containing recombinant mouse granulocyte-macrophage colony-stimulating factor (GM-CSF) (20 ng/mL, BioLegend). Fresh media with recombinant mouse GM-CSF were added into the culture on day 3. The immature BMDCs were collected and ready to use on day 7. For confocal microscopy, BMDCs were seeded overnight on round 10 mm coverslips in a 12-well plate at 1 × 10^5 cells per well. Cells were treated with

fluorescein-cGAMP in various formulations at 1 μg/mL for the indicated time. All samples were mounted with mounting solution containing DAPI (Vector Laboratories, Burlingame, CA) to stain the nuclei and were imaged using a fluorescent microscope. For *in vitro* gene expression studies, BMDCs were plated overnight at 5×10^5 cells per well of a 6-well tissue culture plates and treated with various formulations of cGAMP for 4 h. Then the cells were used for *in vitro* gene expression studies as described previously.

Immunofluorescence

To investigate the entry of hNVs into target cells, B16F10 were plated into glass bottom dishes. After overnight culture, the cells were labeled with DiD and then incubated with DiO-labeled hNVs for 24 h. After being stained with DAPI, the cells were finally observed under the CLSM.

***In vivo* evaluation of single NVs loaded with cGAMP**

4T1 tumor recurrence model was developed as described above. After surgery, the mice received three doses of i.v. injection of PBS, hNVs, cGAMP, P-NVs@cGAMP, M1-NVs@cGAMP, SαV-C-NVs@cGAMP, or hNVs@cGAMP (300 μg of NVs and 36 μg of cGAMP per mouse) every other day. Tumor volume was measured and animals were treated as described above.

Supplementary References

1. Rao, L., et al. Cancer Cell Membrane-Coated Upconversion Nanoprobes for Highly Specific Tumor Imaging. *Adv. Mater.* **28**, 3460-3466 (2016).

2. Deng, L., et al. STING-Dependent Cytosolic DNA Sensing Promotes Radiation-Induced Type I Interferon-Dependent Antitumor Immunity in Immunogenic Tumors. *Immunity* **41**, 843-852 (2014).

3. Liu, Z., et al. Hypofractionated EGFR Tyrosine Kinase Inhibitor Limits Tumor Relapse Through Triggering Innate and Adaptive Immunity. *Sci. Immunol.* **4**, eaav6473 (2019).
