# Supplementary material for: Hybrid cellular membrane nanovesicles amplify macrophage immune responses against cancer recurrence and metastasis
Source: Nat Commun. 2020 Sep 30;11:4909. doi: 10.1038/s41467-020-18626-y (PMC7527506; doi:10.1038/s41467-020-18626-y)
Supplement: Supplementary file 3 — Reporting Summary [file 41467_2020_18626_MOESM3_ESM.pdf]

nature research

Corresponding author(s): Xiaoyuan Chen

Last updated by author(s): Aug 18, 2020

Reporting Summary

Nature Research wishes to improve the reproducibility of the work that we publish. This form provides structure for consistency and transparency in reporting. For further information on Nature Research policies, see Authors & Referees and the Editorial Policy Checklist .

Statistics

For all statistical analyses, confirm that the following items are present in the figure legend, table legend, main text, or Methods section.

n/a	Confirmed	
☐	☒	The exact sample size (n) for each experimental group/condition, given as a discrete number and unit of measurement
☐	☒	A statement on whether measurements were taken from distinct samples or whether the same sample was measured repeatedly
☐	☒	The statistical test(s) used AND whether they are one- or two-sided<br>*Only common tests should be described solely by name; describe more complex techniques in the Methods section.*
☐	☒	A description of all covariates tested
☐	☒	A description of any assumptions or corrections, such as tests of normality and adjustment for multiple comparisons
☐	☒	A full description of the statistical parameters including central tendency (e.g. means) or other basic estimates (e.g. regression coefficient) AND variation (e.g. standard deviation) or associated estimates of uncertainty (e.g. confidence intervals)
☐	☒	For null hypothesis testing, the test statistic (e.g. F, t, r) with confidence intervals, effect sizes, degrees of freedom and P value noted<br>*Give P values as exact values whenever suitable.*
☒	☐	For Bayesian analysis, information on the choice of priors and Markov chain Monte Carlo settings
☒	☐	For hierarchical and complex designs, identification of the appropriate level for tests and full reporting of outcomes
☒	☐	Estimates of effect sizes (e.g. Cohen's d, Pearson's r), indicating how they were calculated

Our web collection on statistics for biologists contains articles on many of the points above.

Software and code

Policy information about availability of computer code

Data collection	CytExpert software 2.0, Microsoft Office Excel 2007
Data analysis	All statistical analyses were performed on Graphpad Prism 5.0. All flowcytometry data were analyzed on CytExpert software 2.0. Bioluminescent and fluorescent images were analyzed on Living image software 4.5.5.

For manuscripts utilizing custom algorithms or software that are central to the research but not yet described in published literature, software must be made available to editors/reviewers. We strongly encourage code deposition in a community repository (e.g. GitHub). See the Nature Research guidelines for submitting code & software for further information.

Data

Policy information about availability of data

All manuscripts must include a data availability statement. This statement should provide the following information, where applicable:
- Accession codes, unique identifiers, or web links for publicly available datasets
- A list of figures that have associated raw data
- A description of any restrictions on data availability

The authors declare that all data supporting the results in this study are available in the paper and Supplementary Information. Source data are available from the corresponding authors upon reasonable request.

Field-specific reporting

Please select the one below that is the best fit for your research. If you are not sure, read the appropriate sections before making your selection.

Life sciences study design

All studies must disclose on these points even when the disclosure is negative.

Sample size	No sample size calculation was performed. Instead, we relied on journal guidelines for a minimum of n = 4 for in vitro and some in vivo tests. For antitumor efficacy and immunology assay, 5-8 animals per group were used. In addition, we adhered to sample size requirements necessary for determining statistical significance.
Data exclusions	No data were excluded.
Replication	Experiments were repeated and experimental findings were reproducible. All the experiments were performed with at least 3 replicates.
Randomization	All samples/organisms were randomly allocated into experimental groups.
Blinding	Bioluminescence imaging were conducted by an independent operator, who was unaware of the treatment conditions. Immunofluorescence slides and images were coded by a lab member who was not involved in this study. Mice were analyzed in a blinded fashion. Mice were tagged with a code after transplantation and all the investigators were blinded during harvesting of tissues and data recording. For all in vitro experiments, the lead investigator was aware of the treatment group as it was very important to know clear difference between samples for downstream assays.

Reporting for specific materials, systems and methods

We require information from authors about some types of materials, experimental systems and methods used in many studies. Here, indicate whether each material, system or method listed is relevant to your study. If you are not sure if a list item applies to your research, read the appropriate section before selecting a response.

Materials & experimental systems

n/a	Involved in the study	
☐	☒	Antibodies
☐	☒	Eukaryotic cell lines
☒	☐	Palaeontology
☐	☒	Animals and other organisms
☒	☐	Human research participants
☒	☐	Clinical data

Methods

n/a	Involved in the study	
☒	☐	ChIP-seq
☐	☒	Flow cytometry
☒	☐	MRI-based neuroimaging

Antibodies

Antibodies used	All the antibodies were provided in the method section. CD11b (Biolegend, FITC, clone M1/70), CD11c (eBioscience, FITC, clone N418), CD206 (Biolegend, APC, clone C068C2), CD3 (eBioscience, FITC, clone 145-2C11), CD4 (eBioscience, FITC, clone GK1.5), CD45 (eBioscience, PerCP-Cyanine5.5, clone 30-F11), CD8 (Biolegend, APC, clone 53–6.7), CD80 (eBioscience, PE, clone 16-10A1), CD86 (eBioscience, APC, clone GL-1), F4/80 (Biolegend, PE-Cyanine7, clone BM8), Ly6C (eBioscience, APC, clone HK1.4), Ly6G (Biolegend, PE, clone 1A8), PE-anti-CD45 (B&D Biosciences), FITC-anti-Cytokeratin (B&D Biosciences), CD61 (Abcam, ab119992), CD14 (Abcam, ab221678), and Melan-A (Abcam, ab210546).
Validation	All antibodies were verified by the supplier and each lot has been quality tested.

Eukaryotic cell lines

Policy information about cell lines

Cell line source(s)	B16F10 murine melanoma, 4T1 murine mammary carcinoma, and 293T human embryonic kidney cell lines were all purchased from the American Type Culture Collection (ATCC). Luciferase-tagged B16F10 and 4T1 cells were established by transfection of B16F10 and 4T1 cells with vectors carrying luciferase and puromycin resistance gene. For construction of SIRPα variant-engineered cells, the cells were sorted and sub-cloned after being transduced by lentivirus expressing cell membrane bound SαV (SαV is an engineered high-affinity SIRPα variant fused with murine SIRPα transmembrane domain).
Authentication	The cell line B16F10, 4T1, and 293T were certified according to the information of surface makers and morphology provided by the manufacturers. Transfected cell lines were verified using positive or negative controls according to manufacturers' suggestions and compared with the data provided by manufacturers.
Mycoplasma contamination	All cell lines were tested for mycoplasma contamination. No mycoplasma contamination was found.
Commonly misidentified lines (See ICLAC register)	No commonly misidentified cell lines were used.

Animals and other organisms

Policy information about studies involving animals; ARRIVE guidelines recommended for reporting animal research

Laboratory animals	C57BL/6 mice (female, 6-10 weeks) and BALB/c mice (female, 6-10 weeks) were both purchased from Hunan Silaike Jinda Laboratory Animal Co. Ltd. (China).
Wild animals	The study did not involve wild animals.
Field-collected samples	The study did not involve samples collected from field.
Ethics oversight	The animal study was approved by the Institutional Review Board of Wuhan University in accordance with the guidelines for the protection of animal subjects.

Note that full information on the approval of the study protocol must also be provided in the manuscript.

Flow Cytometry

Plots

Confirm that:

- [x] The axis labels state the marker and fluorochrome used (e.g. CD4-FITC).
- [x] The axis scales are clearly visible. Include numbers along axes only for bottom left plot of group (a 'group' is an analysis of identical markers).
- [x] All plots are contour plots with outliers or pseudocolor plots.
- [x] A numerical value for number of cells or percentage (with statistics) is provided.

Methodology

Sample preparation	For tissue sample, the tissue was first mechanically disrupted from mice and divided into small pieces and homogenized in cold staining buffer to form single cell suspensions in the presence of digestive enzyme.
Instrument	CytoFLEX flow cytometer (Beckman)
Software	CytoExpert software (Beckman)
Cell population abundance	No sorting was performed.
Gating strategy	Gating strategies are referred to those described in the BD website (https://www.bio-rad-antibodies.com/flow-cytometry-gating-strategies.html). Gating was first based on FSC/SCC together with viability dyes and singlet populations. The cell populations within the gate were further analyzed based on the expression of markers.

- [x] Tick this box to confirm that a figure exemplifying the gating strategy is provided in the Supplementary Information.
